# Long-Term Risk of Recurrent Cervical Artery Dissection and Stroke After Pregnancy

**DOI:** 10.1001/jamanetworkopen.2025.21539

**Published:** 2025-07-17

**Authors:** Sandro K. Fischer, Josefin E. Kaufmann, Tiina M. Metso, Turgut Tatlisumak, Johannes Wischmann, Lars Kellert, Lukas Mayer-Suess, Michael Knoflach, Regina von Rennenberg, Christian H. Nolte, Cheyenne Lee, Chad M. Aldridge, Bradford B. Worrall, Andrew T. Weko, Andrew M. Southerland, Judea P. Wiggins, Jennifer J. Majersik, Philipp Baumgartner, Susanne Wegener, Issa Metanis, Ronen R. Leker, Vanessa Cano-Nigenda, Antonio Arauz, Anabella Frances, Ignacio Bozas, Juan J. Martin, Annaelle Zietz, Alexandros Polymeris, Valerian L. Altersberger, Giorgia Abrignani, Paola Castellini, Antonio Genovese, Lilia Latte, Maria Claudia Trapasso, Marialuisa Zedde, Anna Bersano, Giulia Marinoni, Giorgio Silvestrelli, Claudio Baracchini, Francesco Favruzzo, Maurizio Paciaroni, Alessandra Spalloni, Rosalba Patella, Manuel Cappellari, Francesco Valletta, Massimo Del Sette, Davide Sassos, Mauro Gentile, Mauro Magoni, Massimo Gamba, Marina Padroni, Cristiano Azzini, Elisa Giorli, Fabio Melis, Rossana Tassi, Rocco Salvatore Calabrò, Valeria Piras, Maurizio Melis, Alessia Giossi, Sandro Sanguigni, Marina Mannino, Valeria Bignamini, Alessandra Gaiani, Alessandro Adami, Rita Bella, Rosario Pascarella, Philippe Lyrer, Henrik Gensicke, Alessandro Pezzini, Christopher Traenka, Stefan T. Engelter

**Affiliations:** 1 Neurology and Neurorehabilitation, University Department of Geriatric Medicine Felix Platter, University of Basel, Basel, Switzerland; 2 Department of Neurology and Stroke Center, Department of Clinical Research, University Hospital Basel, University of Basel, Basel, Switzerland; 3 Department of Neurology, Helsinki University Central Hospital, Helsinki, Finland; 4 Department of Neuroscience, Institute of Neuroscience and Physiology, Sahlgrenska Academy, University of Gothenburg, Gothenburg, Sweden; 5 Department of Neurology, Sahlgrenska University Hospital, Gothenburg, Sweden; 6 Department of Neurology, Ludwig Maximilian University Hospital, Ludwig Maximilian University of Munich, Munich, Germany; 7 Department of Neurology, Medical University of Innsbruck, Innsbruck, Austria; 8 Department for Neurology and Experimental Neurology and Center for Stroke Research Berlin, Charité Universitätsmedizin, Berlin, Germany; 9 Berlin Institute of Health, Charité Universitätsmedizin, Berlin, Germany; 10 Department of Neurology, University of Virginia, Charlottesville; 11 Department of Public Health Sciences, University of Virginia, Charlottesville; 12 Department of Neurology, University of Utah, Salt Lake City; 13 Department of Neurology, University Hospital Zurich, University of Zurich, Zurich, Switzerland; 14 Hadassah-Hebrew University Medical Center, Jerusalem, Israel; 15 Stroke Clinic, Instituto Nacional de Neurología y Neurocirugía Manuel Velasco Suárez, Mexico City, Mexico; 16 Stroke Unit Sanatorio Allende Cordoba, Argentina; 17 Programma Stroke Care, Dipartimento di Emergenza-Urgenza, Azienda Ospedaliera Universitaria, Parma, Italia; 18 Neurology Unit, Stroke Unit, Azienda Unità Sanitaria Locale-IRCCS di Reggio Emilia, Italia; 19 S.C. Malattie Cerebrovascolari, Fondazione IRCCS Istituto Neurologico “Carlo Besta,” Milan, Italy; 20 Stroke Unit, Dipartimento di Neuroscienze, Ospedale Carlo Poma, Mantua, Italy; 21 U.O.S.D. Stroke Unit e Laboratorio di Neurosonologia, Azienda Ospedale-Università di Padova, Padua, Italy; 22 Department of Neurosciences and Rehabilitation, University of Ferrara, Ferrara, Italy; 23 Stroke Unit, Azienda Ospedaliera Sant’Andrea, Università “La Sapienza,” Rome, Italy; 24 Stroke Unit, Azienda Ospedaliera Universitaria Integrata Borgo Trento, Verona, Italy; 25 IRCCS Ospedale Policlinico San Martino, Genoa, Italy; 26 IRCCS Istituto delle Scienze Neurologiche di Bologna, UOC Neurologia e Rete Stroke Metropolitana, Ospedale Maggiore, Bologna, Italy; 27 Stroke Unit, Neurologia Vascolare, ASST Spedali Civili di Brescia, Brescia, Italy; 28 U.O. Neurologia, Stroke Unit, Azienda Ospedaliera Universitaria S. Anna, Ferrara, Italy; 29 U.O. Neurologia, Ospedale S. Andrea, La Spezia, Italy; 30 S.S. NeuroVascolare Ospedale Maria Vittoria, ASL Città di Torino, Torino, Italy; 31 U.O.C. Stroke Unit, Azienda Ospedaliera Universitaria Senese, Siena, Italy; 32 IRCCS Centro Neurolesi Bonino-Pulejo, Messina, Italy; 33 S.C. Neurologia e Stroke Unit, Dipartimento Neuroscienze e Riabilitazione, Azienda Ospedaliera “G. Brotzu,” Cagliari, Italy; 34 U.O. Neurologia, Istituti Ospitalieri, ASST Cremona, Cremona, Italy; 35 Dipartimento di Neurologia, Ospedale “Madonna del Soccorso,” San Benedetto del Tronto, Italy; 36 Stroke Unit, Ospedale Civico, Palermo, Italy; 37 Stroke Unit, U.O Neurologia, Ospedale “S. Chiara,” APSS Trento, Trento, Italy; 38 Stroke Center, Dipartimento di Neurologia, IRCSS Sacro Cuore Negrar, Verona, Italy; 39 Dipartimento di Scienze Mediche, Chirurgiche e Tecnologie Avanzate, Università di Catania, Catania, Italy; 40 Santa Maria della Misericordia Hospital, AULSS5 Polesana, Rovigo, Italy; 41 Dipartimento di Medicina e Chirurgia, Università degli Studi di Parma, Parma, Italy

## Abstract

**Importance:**

Cervical artery dissection (CeAD) is a leading cause of stroke in young people and can occur during pregnancy. In women with prior CeAD, it is unclear whether future pregnancies increase the risk of recurrent CeAD, stroke, or death.

**Objective:**

To compare the frequency of recurrent CeAD, stroke, or death as a composite outcome between patients with CeAD who became pregnant vs those who did not.

**Design, Setting, and Participants:**

The Long-Term Risk of Recurrent Cervical Artery Dissection and Stroke After Pregnancy (LONG-RECAP) study was an international, multicenter, registry-based, explorative cohort study with data from May 1, 1990, and April 30, 2023, among patients with CeAD at 33 stroke centers across 9 countries who had at least 6 months of follow-up for whom detailed information was available on absence vs presence of subsequent pregnancies and the occurrence of outcome events.

**Exposure:**

Pregnancy after CeAD.

**Main Outcomes and Measures:**

Composite of recurrent CeAD, stroke (any type), and death (all causes) during follow-up at least 6 months after initial CeAD. Secondary outcomes were the components of the composite outcome. Cox proportional hazards regression analysis with and without adjustment for age was used to examine the association between pregnancy and outcomes.

**Results:**

Among 1013 female patients with CeAD (median [IQR] age, 42 [35-48] years), 114 (11.3%) became pregnant during a median (IQR) follow-up of 5.3 (2.0-11.3) years. The composite outcome occurred in 10 of 114 patients (8.8%), including 7 patients with recurrent CeADs, 2 with ischemic strokes, and 1 with intracerebral hemorrhage, in the pregnancy group compared with 65 of 899 patients (7.2%), including 32 with recurrent CeAD, 26 with ischemic strokes, 4 with intracerebral hemorrhages, and 5 deaths (2 patients had 2 outcomes), in the nonpregnancy group (unadjusted hazard ratio, 1.08; 95% CI, 0.56-2.08; age-adjusted hazard ratio, 0.77; 95% CI, 0.38-1.56). Five of 10 events in the pregnancy group occurred post partum (4 CeADs and 1 stroke). Age-adjusted hazard ratios for secondary outcomes were 1.03 (95% CI, 0.43-2.46) for recurrent CeAD, 0.53 (95% CI, 0.13-2.12) for ischemic stroke, 1.01 (95% CI, 0.11-9.32) for intracerebral hemorrhage, and 0.90 (95% CI, 0.03-24.88) for death.

**Conclusions and Relevance:**

In this cohort study of women with a history of CeAD, becoming pregnant was not associated with an increased risk of recurrent CeAD, stroke, or death. These findings may be helpful for individual counseling and family planning for women with prior CeAD.

## Key Points

**Question:**

Is pregnancy associated with increased risk of a composite outcome of recurrent cervical artery dissection (CeAD), ischemic stroke, intracerebral hemorrhage, or death (all causes) in women with a history of CeAD?

**Findings:**

In this multicenter, registry-based cohort study of 1013 female patients with CeAD, 11.3% became pregnant during a median follow-up time of 5.3 years. The frequency of the composite outcome did not differ significantly between women who got pregnant and those who did not.

**Meaning:**

These findings suggest that pregnancy is not associated with an increased risk of subsequent events; therefore, prior CeAD does not justify advising against future pregnancy.

## Teaser

This cohort study of female patients with cervical artery dissection (CeAD) examines whether future pregnancies are associated with increased risk of recurrent CeAD, stroke, or death.

## Introduction

Cervical artery dissection (CeAD) is a leading cause of stroke in young people,^1,2^ including women of childbearing age.^3,4,5,6,7^ Hormonal alteration and mechanical strain due to increase in cardiac output and blood volume may increase the risk of CeAD during pregnancy and the postpartum period.^8,9,10^ Recent studies have found that pregnancy, in particular the postpartum period, was associated with the occurrence of CeAD^5^ and that CeAD is a rare but important complication of pregnancy and the postpartum period.^7^ Studies^4,6^ specifically investigating the risks of CeAD recurrence or stroke in women with prior CeAD who became pregnant thereafter are scarce and based on small study samples. Thus, in clinical practice, it remains unclear how to counsel women with prior CeAD who are considering pregnancy. Therefore, we designed a large, international, multicenter, registry-based cohort study comparing the frequency of recurrent CeAD, stroke, or death among patients with CeAD who became pregnant after a prior CeAD vs those who did not.

## Methods

### Study Population

This retrospective, registry-based cohort study analyzed pooled individual patient data from 33 international stroke centers (9 countries) with special interest and expertise in the diagnosis and treatment of patients with CeAD^11,12^ (see eTable 1 in Supplement 1 for a full list of contributing centers). The pooling of data was in accordance with the individual legal and ethical requirements of the respective centers. All centers followed respective national rules of approval and informed consent of the included patients. The study in Basel was approved by the local authorities and ethics committee. The Long-Term Risk of Recurrent Cervical Artery Dissection and Stroke After Pregnancy (LONG-RECAP) study is registered with ClinicalTrials.gov^13^ and followed the Strengthening the Reporting of Observational Studies in Epidemiology (STROBE) guidelines for reporting results from cohort studies.^14^

For this study, each center provided individual patient data on consecutive patients with CeAD from their prospectively ascertained registries of patients with CeAD between May 1, 1990, and April 30, 2023. Data pooling for the current analysis was performed between January 1 and December 15, 2023. Eligibility criteria were female sex and long-term follow-up data (from at least 6 months to the longest follow-up available) on the presence vs absence of the following outcome events: recurrent CeAD; ischemic stroke; intracerebral hemorrhage and death (all causes), applying criteria used in prior research^15^; and presence vs absence of pregnancy during follow-up. Because data collection focused on variables most relevant to the study objectives, information on race and ethnicity was not collected. Contributing centers applied the same widely accepted and previously published diagnostic CeAD criteria.^16,17^

The study compared patients with CeAD who became pregnant after their initial CeAD (pregnancy group) with those who did not become pregnant (nonpregnancy group). The likelihoods of becoming pregnant and of recurrent dissection decrease with advancing age.^18,19^ In addition, there is an increase in maternal age at childbirth and an increasing use of medically assisted reproduction.^20^ Moreover, in the literature there is no universally accepted threshold of the upper end of childbearing age.^4,5,6,21^ Thus, during the design period of our study, we considered it more appropriate to adjust for the variable age rather than to set an upper age limit for women of childbearing age in the primary analyses and to add sensitivity analyses applying literature-reported age thresholds.

### Study Variables

The following baseline data were obtained from the participating centers’ registries with definitions and criteria applied in prior research^22,23^: age, sex, site of dissection (internal carotid artery and/or vertebral artery, or multiple), occlusion vs nonocclusion of the dissected artery, single- vs multiple-vessel dissection, presentation with ischemic symptoms (ie, ischemic stroke including retinal infarction, and transient ischemic attack, including amaurosis fugax, with stroke cases confirmed by imaging), presentation with local clinical symptoms, (eg, Horner syndrome and cranial nerve palsy), recent mechanical trigger events to the head or neck,^24^ prior infection in the month preceding the onset of symptoms, history of migraine, known connective tissue disease, initial CeAD occurring during pregnancy, delivery, or postpartum period (applying definitions from prior research^8^), diabetes, hypertension, hypercholesterolemia, and current smoking. In each center, follow-up had been conducted through in-person visits, telephone, mail, or email, supplemented by review of medical records where appropriate.

### Exposure Variable

Pregnancy after CeAD (the exposure variable in this study) was defined as the time from the onset of a pregnancy (ie, presumed conception) until the end of the postpartum period, defined as 6 weeks after delivery as done in prior research.^3,8,25^ Because other research defined the postpartum period as up to 12 weeks after delivery,^26^ data on outcomes occurring between 6 and 12 weeks post partum were provided separately. The following pregnancy characteristics were obtained: (1) number of pregnancies since the CeAD, (2) dichotomized type of pregnancy (ie, completed pregnancy vs miscarriages or abortions), (3) the date of delivery (or time point of delivery in months after CeAD), and (4) mode of delivery (cesarean vs vaginal).

### Outcome Events

The main outcome was a composite of (1) recurrent CeAD, (2) ischemic stroke or intracerebral hemorrhage with confirmation by neuroimaging (applying criteria used in prior research),^15^ and (3) death of all causes. Secondary outcomes were the individual components of the composite outcome. When a patient had more than 1 outcome event, only the first outcome was counted for the total number of composite outcomes. Outcome data were collected during follow-up visits and supplemented by a review of medical records. For each patient with composite outcomes, whether the outcome occurred while receiving antithrombotic therapy (ie, any antiplatelet agent or anticoagulant) or not was recorded. In each patient, data from the longest follow-up assessment available was used. In the pregnancy group, each outcome was identified as occurring (1) before pregnancy, (2) during pregnancy, or (3) after pregnancy (>6 weeks after delivery). For events occurring during pregnancy, events were assigned to the following 3 pregnancy phases: (1) pregnancy sensu strictu (ie, conception until delivery), (2) delivery, and (3) postpartum period.

### Statistical Analysis

We compared patients with vs without pregnancy after the initial CeAD. First, we examined differences in baseline characteristics between women with and without pregnancy after CeAD. In this step, we used the χ^2^ test, Fisher exact test, or Wilcoxon rank-sum test as appropriate and univariable logistic regression. Second, we used penalized Cox proportional hazards regression analysis to calculate hazard ratios (HRs) to examine the association between pregnancy during follow-up and the main and secondary outcomes. HRs for secondary outcomes were calculated individually. We performed unadjusted and adjusted Cox proportional hazards regression analyses using the penalized likelihood method to account for low event rates.^27^ We adjusted our analyses by the age at initial CeAD in the multivariable analyses. Third, we performed time-to-event analysis to estimate the cumulative incidence function for demonstration of CeAD recurrence during follow-up, separately for women with and without pregnancy after initial CeAD. Cumulative incidence curves were compared using the log-rank test.

Continuous variables are presented as medians (IQRs) and categorical variables as numbers (percentages). The relative effects of the Cox proportional hazards regression models are displayed in forest plots, showing HRs with corresponding 95% CIs. Statistical significance was determined at α = .05 in all analyses. All statistical analyses were conducted between December 2023 and April 2025 using the statistical software package R, version 4.4.1 (R Project for Statistical Computing). Post hoc we also compared the event rates during pregnancy and post partum to the time before and thereafter. We conducted sensitivity analyses using 3 upper age limits for patients at the time of at initial CeAD: 42 years or younger,^4,6^ 45 years or younger,^5^ and 49 years or younger.^21^ This approach applied the age limits proposed by the World Health Organization^21^ and used in prior research^4,5,6^ on the topic. For each subgroup, we repeated all analyses, including the Cox proportional hazards regression model for the main outcome. A 2-sided *P* < .05 was considered statistically significant.

## Results

### Study Population and Baseline Characteristics

Among 1149 women with a history of CeAD, 1013 (88.2%) were eligible for analyses. The reasons the patients were excluded were insufficient follow-up time (ie, <6 months; n = 78), missing data on pregnancies (n = 45), missing data on outcome events (n = 8), or regulatory reasons (n = 5) (eFigure 1 in Supplement 1). Of the 1013 included patients, 114 (11.3%) had at least 1 pregnancy after the initial CeAD (pregnancy group), whereas 899 patients (88.7%) did not get pregnant during follow-up and served as the comparison group.

Baseline characteristics of the study patients are given in Table 1. In brief, the median (IQR) age of study patients at time of the initial CeAD was 42 (35-48) years. Dissection of the internal carotid artery occurred in 629 patients (62.3%), and 154 patients (15.2%) had multiple dissections. A total of 659 patients (65.2%) presented with an ischemic stroke, 139 (13.7%) with transient ischemic attack, and 243 (24.0%) with local symptoms only.

**Table 1.** Baseline Characteristics of the Study Patients

Characteristic	No./total No. (%) of patients			Unadjusted *P* value	Unadjusted OR (95% CI)
	All women (N = 1013)	Women with pregnancy after initial CeAD (n = 114)	Women without pregnancy after initial CeAD (n = 899)		
Age at initial CeAD, median (IQR), y	42 (35-48)	31 (27-36)	43 (37-49)	<.001	NA
Site of CeAD					
Internal carotid artery	629/1012 (62.3)	68/114 (59.6)	561/898 (62.5)	.63	0.89 (0.60-1.33)
Vertebral artery	431/1012 (42.5)	56/114 (49.1)	375/898 (41.8)	.16	1.35 (0.91-1.99)
Multivessel dissection^a^	154/1012 (15.2)	21/114 (18.4)	133/898 (14.8)	.38	1.30 (0.76-2.12)
Occlusion	326/1009 (32.3)	33/113 (29.2)	293/896 (32.7)	.52	0.85 (0.55-1.29)
Clinical presentation at baseline					
Pure local symptoms^b^	243/1012 (24.0)	28/114 (21.6)	215/898 (23.9)	.97	1.04 (0.65-1.61)
Ischemic stroke^b^	659/1011 (65.2)^c^	72/114 (63.2)^c^	587/897 (65.4)^c^	.71	0.91 (0.61-1.37)
TIA ^b^	139/1012 (13.6)^c^	18/114 (15.8)^c^	121/898 (13.5)^c^	.60	1.20 (0.68-2.02)
Predisposing factors					
Mechanical trigger event^d^	192/983 (19.5)	34/112 (30.4)	158/871 (18.1)	.003	1.99 (1.27-3.06)
Recent infection^d^	123/986 (12.5)	15/109 (13.8)	108/877 (12.3)	.78	1.14 (0.61-1.98)
Migraine	376/1007 (37.3)	42/113 (37.2)	334/894 (37.4)	>.99	0.99 (0.66-1.48)
Connective tissue disease	37/958 (3.9)	5/105 (4.8)	32/853 (3.8)	.81	1.28 (0.43-3.09)
Associated with pregnancy	27/927 (2.9)	6/102 (5.9)	21/825 (2.5)	.11	2.39 (0.86-5.74)
Vascular risk factors					
Hypertension	220/1008 (21.8)	14/114 (12.3)	206/894 (23.0)	.01	0.47 (0.25-0.81)
Diabetes	28/1008 (2.8)	2/114 (1.8)	26/894 (2.9)	.69	0.60 (0.10-2.03)
Hypercholesterolemia	147/1007 (14.6)	9/114 (7.9)	138/893 (15.5)	.04	0.47 (0.22-0.90)
Active smoker	233/1004 (23.2)	25/114 (21.9)	208/890 (23.4)	.82	0.92 (0.57-1.45)

Abbreviations: CeAD, cervical artery dissection; NA, not applicable; OR, odds ratio; TIA, transient ischemic attack.

^a^ Multivessel dissection includes patients with internal carotid artery plus vertebral artery dissection as well as bilateral internal carotid artery dissection and bilateral vertebral artery dissection.

^b^ A total of 22 patients (2.2%) did not present any symptoms or cerebral ischemia at baseline.

^c^ A total of 52 patients (5.1%) had both a TIA and an ischemic stroke as presenting symptoms.

^d^ In the month preceding the onset of initial CeAD.

Compared with the nonpregnancy group, patients in the pregnancy group were younger (median [IQR] age, 31 [27-36] vs 43 [37-49] years), more often had mechanical trigger events (34 of 112 [30.4%] vs 158 of 871 [18.1%]), and less often had hypertension (14 of 114 [12.3%] vs 206 of 894 [23.0%]) or hypercholesterolemia (9 of 114 [7.9%] vs 138 of 893 [15.5%]). Other baseline characteristics did not differ between the groups (Table 1).

### Outcomes

**Figure 1.**
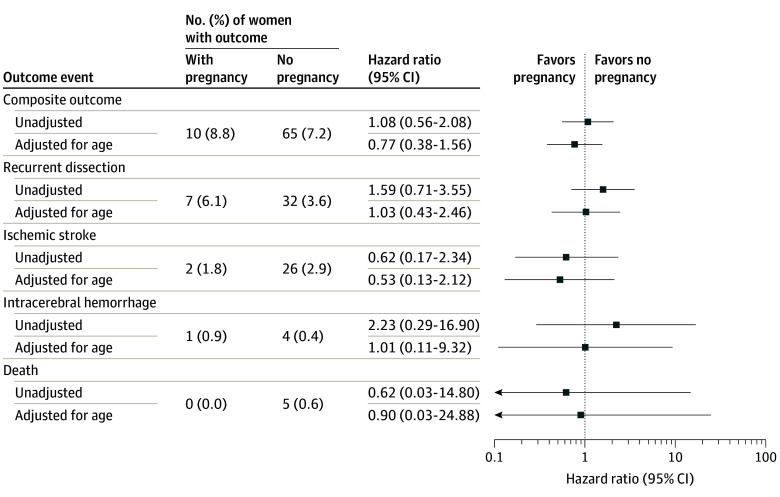
Main and Secondary Outcomes

The main outcome (composite of recurrent CeAD, ischemic stroke, intracerebral hemorrhage, or all-cause death) was observed in a total of 75 of 1013 women (7.4%), including 10 of 114 women (8.8%) in the pregnancy group and 65 of 899 women (7.2%) in the nonpregnancy group (Table 2). The unadjusted HR was 1.08 (95% CI, 0.56-2.08). Adjustment for age resulted in an adjusted HR of 0.77 (95% CI, 0.38-1.56) (Figure 1). Median (IQR) follow-up time of all women was 5.3 (2.0-11.2) years.

**Table 2.** Main and Secondary Outcomes

Outcome	No. (%) of patients			Unadjusted *P* value	HR (95% CI)	
	All women (N = 1013)	Women with pregnancy after initial CeAD (n = 114)	Women without pregnancy after initial CeAD (n = 899)		Unadjusted	Adjusted for age^a^
Composite outcome	75 (7.4)^c^	10 (8.8)	65 (7.2)^c^	.69	1.08 (0.56-2.08)	0.77 (0.38-1.56)
Receiving antithrombotic therapy	39 (52.0)	6 (60)	33 (50.8)	NA	NA	NA
Without antithrombic therapy	13 (17.3)	3 (30)	10 (15.4)	NA	NA	NA
Antithrombotic therapy unknown^d^	23 (30.7)^d^	1 (10)	22 (33.8)^d^	NA	NA	NA
Recurrent dissection	39 (3.8)	7 (6.1)^b^	32 (3.6)^c^	.19	1.59 (0.71-3.55)	1.03 (0.43-2.46)
Asymptomatic	5 (12.8)	0	5 (15.6)	.56	NA	NA
Pure local symptoms	19 (48.7)	3 (42.9)	16 (50.0)	>.99	NA	NA
Ischemic stroke	10 (25.6)	2 (28.6)^b^	8 (25.0)	>.99	NA	NA
TIA	6 (15.4)	3 (42.9)^b^	3 (9.4)	.06	NA	NA
Ischemic stroke (independent of recurrent CeAD)	28 (2.8)	2 (1.8)	26 (2.9)^c^	.76	0.62 (0.17-2.34)	0.53 (0.13-2.12)
Intracerebral hemorrhage	5 (0.5)	1 (0.9)	4 (0.4)^c^	.45	2.23 (0.29-16.90)	1.01 (0.11-9.32)
Death	5 (0.5)	0	5 (0.6)^c^	>.99	0.62 (0.03-24.88)	0.90 (0.03-24.88)
Follow-up time, median (IQR), y	5.25 (2.00-11.25)	7.21 (3.94-12.00)	5.00 (1.92-10.83)	.003	NA	NA

Abbreviations: CeAD, cervical artery dissection; HR, hazard ratio; NA, not applicable; TIA, transient ischemic attack.

^a^ Hazard ratios were adjusted for age at initial CeAD.

^b^ One patient in the pregnancy group experienced both TIA and ischemic stroke due to recurrent CeAD.

^c^ Two patients in the nonpregnancy group experienced 2 outcome events each (recurrent dissection plus intracerebral hemorrhage and ischemic stroke plus death).

^d^ For 4 of 5 patients who died, antithrombotic therapy at time of death is unknown.

During a total follow-up of 7393 patient-years, we observed 75 main outcome events (composite of 39 recurrent CeADs, 28 ischemic strokes, 5 intracerebral hemorrhages, and 5 deaths [2 patients had 2 outcome events]), which translates to a primary event rate of 1.0% per patient-year. The event rate was 1.1% per patient-year in the pregnancy group and 1.0% per patient-year in the nonpregnancy group.

In the pregnancy group, the 10 patients with main outcomes included 7 patients with recurrent CeAD (of whom 4 presented with cerebral ischemia and 3 with local symptoms only), 2 ischemic stroke (not associated with CeAD), 1 intracerebral hemorrhage, and no deaths (Table 2). Five of the 10 patients with primary outcomes had events during pregnancy. Among these 5 patients (with events during pregnancy), 4 patients had recurrent CeAD that occurred during the 6-week postpartum period (Table 3), whereas 1 patient experienced an ischemic stroke due to large artery atherosclerosis, also during the 6-week postpartum period. No outcome events occurred between 6 and 12 weeks post partum. To summarize, 4 of 114 women (3.5%) in the pregnancy group experienced a recurrent CeAD, all occurring in the postpartum period. Detailed individual patient characteristics of the women with postpartum recurrent CeAD are provided in eTable 2 in Supplement 1. Among the 5 other patients in the pregnancy group, who had main outcomes outside the pregnancy period, 2 had a recurrent CeAD before their pregnancy, 1 had a recurrent CeAD after her pregnancy (6 months after delivery), and 2 experienced strokes after their pregnancy (1 ischemic stroke 6 months after delivery and 1 intracerebral hemorrhage 13 years after delivery). The post hoc comparison showed that the event rate was higher during pregnancy plus post partum compared with the time before and after pregnancy (eTable 9 in Supplement 1).

**Table 3.** Pregnancy Characteristics and Recurrent CeAD

Characteristic	All patients who became pregnant (n = 114)	Patients with recurrent CeAD (n = 7)	Patients without recurrent CeAD (n = 107)
Follow-up time, median (IQR), y	7.2 (3.9-12.0)	7.3 (5.0-7.7)^a^	7.2 (4.0-12.5)^a^
Age at baseline, median (IQR), y	31.0 (27.0-36.0)	34.0 (31.0-37.0)^b^	31.0 (27.0-36.0)^b^
Time point of recurrent CeAD, No. (%)			
Before pregnancy	NA	2 (28.6)	NA
During pregnancy sensu strictu		0	
During delivery		0	
During 0-6 wk post partum		4 (57.1)	
During 6-12 wk post partum		0	
After postpartum period (>12 wk after delivery)		1 (14.3)	
No. of pregnancies during follow-up^c^	158	11	147
Completed pregnancies	122	9	113
Abortions or miscarriages	36	2	34
Mode of delivery of completed pregnancies, No. (%)			
Vaginal	50/122 (41.0)	4/9 (44.4)	46/113 (40.7)
Cesarean	72/122 (59.0)	5/9 (55.6)	67/113 (59.3)

Abbreviations: CeAD, cervical artery dissection; NA, not applicable.

^a^ Unadjusted *P* = .64 for follow-up time.

^b^ Unadjusted *P* = .25 for age.

^c^ Missing data on number of pregnancies for 1 patient and on number of abortions for 9 patients.

In the nonpregnancy group, 32 patients had recurrent CeAD (of whom 11 presented with cerebral ischemia, 16 presented with local symptoms only, and 5 were asymptomatic); 26 patients had ischemic strokes (unassociated with CeAD), 4 patients had intracerebral hemorrhage, and 5 died (Table 2). Two patients in the nonpregnancy group experienced 2 outcome events each (recurrent CeAD plus intracerebral hemorrhage [n = 1, not related to each other] and ischemic stroke leading to death [n = 1]), whereas none of the patients in the pregnancy group had multiple outcome events. Comparing the 2 groups regarding each component of the composite outcome, the adjusted HRs were 1.03 (95% CI, 0.43-2.46) for recurrent CeAD, 0.53 (95% CI, 0.13-2.12) for ischemic stroke, 1.01 (95% CI, 0.11-9.32) for intracerebral hemorrhage, and 0.90 (95% CI, 0.03-24.88) for death.

### Sensitivity Analyses

Among the subgroups of patients aged 42 years or younger, 45 years or younger, and 49 years or younger when they had had their initial CeAD, the unadjusted HRs of the pregnancy group regarding the composite outcome were 0.82 (95% CI, 0.40-1.67), 0.93 (95% CI, 0.46-1.87), and 1.00 (95% CI, 0.52-1.95) and after adjustment for age were 0.77 (95% CI, 0.36-1.64), 0.72 (95% CI, 0.34-1.54), and 0.77 (95% CI, 0.38-1.57), respectively (eFigures 2-4 and eTables 3-8 in Supplement 1). Applying these age limits led to the exclusion of 11, 10, and 1 women, respectively, who became pregnant during follow-up.

### Pregnancy Characteristics

Among the 114 women who became pregnant, there were 158 total pregnancies (36 miscarriages or abortions and 122 successful deliveries). In our sample, cesarean delivery was the more frequent mode of delivery (72 of 122 [59.0%]). A detailed summary of pregnancy characteristics is presented in Table 3. In women with pregnancy after initial CeAD, follow-up time and age at baseline did not differ significantly between those who experienced recurrent CeAD and those who did not (median [IQR] follow-up time, 7.3 [5.0-7.7] vs 7.2 [4.0-12.5] years; unadjusted *P* = .64; median [IQR] age, 34 [31-37] vs 31 [27-36] years; unadjusted *P* = .25). Frequency of cesarean delivery did not differ between groups (5 of 9 [55.6%] vs 67 of 113 [59.3%]) (Table 3). The 7 women who became pregnant after CeAD and who had recurrent CeAD had 11 pregnancies, of which 9 ended successfully with delivery. Delivery was vaginal in 4 and cesarean in 5 patients, respectively. In the 4 patients with recurrent CeAD during the pregnancy period, mode of delivery of the 4 was vaginal in 3 and cesarean in 1. In none of those 4 patients was their initial CeAD associated with pregnancy. One of those 4 patients had 2 pregnancies after her initial CeAD. Although her first pregnancy was uneventful, her recurrent CeAD occurred during her second pregnancy (Table 3).

### Time to CeAD Event

**Figure 2.**
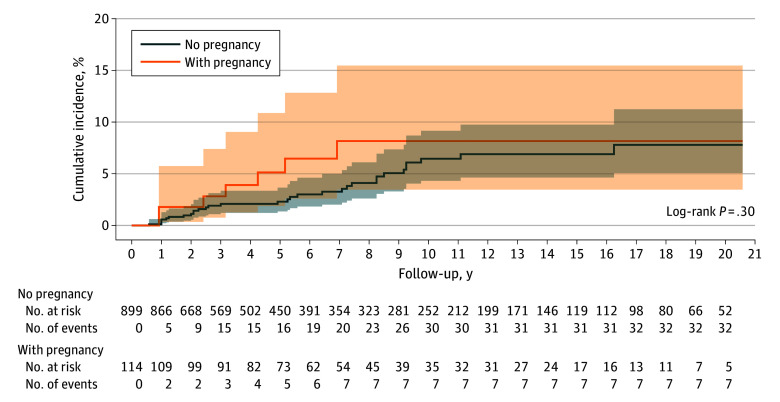
Cumulative Incidence of Recurrent Cervical Artery Dissection During Follow-Up The solid lines represent the estimated cumulative incidence of recurrent cervical artery dissection for each group, with the shaded areas indicating the corresponding 95% CIs.

The overall estimated cumulative rate of recurrence of CeAD in our study was 3.1% (95% CI, 2.0%-4.5%) and is shown in Figure 2. The estimated cumulative rate of recurrence was 1.2% (95% CI, 0.6-2.1) during 2 years and 6.7% (95% CI, 4.7-9.2) during 10 years. Considering the total time at risk of all patients (7393 patient-years), we calculated an incidence rate for recurrent CeAD of 0.53% per patient-year (95% CI, 0.30%-1.5%) for all women. The incidence rate for recurrent CeAD was 0.74% (95% CI, 0.30%-1.52%) per patient-year in the pregnancy group and 0.50% (95% CI, 0.34%-0.70%) per patient-year in the nonpregnancy group.

## Discussion

Our analyses revealed the following key findings. First, in patients with a personal history of CeAD, subsequent pregnancy does not seem to increase the risk of recurrent CeAD, ischemic stroke, intracerebral hemorrhage, or death. Second, in women with a prior CeAD and a subsequent pregnancy, who experience a recurrent CeAD, the postpartum period is the most common period for such an event. Third, our data are insufficient to determine whether the risk of recurrent CeAD is associated with the mode of delivery (ie, vaginal vs cesarean).

It has been unclear so far, whether becoming pregnant increases the risk of a recurrent CeAD in women who previously had CeAD. We are aware of only 2 prior studies that addressed this question: one with 89 patients in whom 18 became pregnant^6^ and one with 53 patients in whom 11 became pregnant.^4^ In both studies,^4,6^ none of the women who became pregnant during follow-up had recurrent CeAD. In this context, it is relevant that among more than 1000 patients (of whom >100 became pregnant), the likelihood of recurrent CeAD, stroke, or death did not differ between those who became pregnant and those who did not.

Interestingly, 4 of 114 patients (3.5%) in the pregnancy group experienced recurrent CeAD during the postpartum period. Thus, our study provides evidence that these events, unlike the observations of the aforementioned small series,^4,6^ indeed happen. The finding that all recurrent CeADs (and one ischemic stroke unrelated to the CeAD) temporally related to a pregnancy took place within the postpartum period suggests that within the entire pregnancy phase the postpartum period seems to be the most vulnerable period for recurrent CeADs. This finding is supported by our post hoc comparison of event rates during pregnancy vs before and thereafter.

These findings are in line with the literature about the occurrence of a first CeAD associated with pregnancy, which reportedly also occurs preferentially within the postpartum period.^5,7,8^ CeAD during pregnancy and within the postpartum period might be due to hormonal alterations and mechanical strain, including blood pressure increases.^8^

Except for younger age, a higher frequency of mechanical triggers, and some differences in vascular risk factor profiles, most baseline characteristics did not differ between the pregnancy and the nonpregnancy group, including the association between pregnancy and initial CeAD. None of the women with a previous pregnancy-related CeAD experienced a recurrent CeAD during any subsequent pregnancy. Thus, our data did not show that there might be a higher risk of a recurrent dissection during a subsequent pregnancy in patients in whom the initial CeAD had occurred during pregnancy.

We observed more than 120 deliveries distributed as vaginal delivery vs cesarean delivery by a rate of 2:3. Nevertheless, our data are insufficient to evaluate whether the mode of delivery was associated with CeAD recurrence because we solely observed 4 recurrent CeADs, which were temporally related to pregnancy. We also do not know the reasons for the choice of delivery and whether that was influenced by a history of prior CeAD.

### Strengths and Limitations

The large sample size, the relatively long observation period, and the multicenter and multinational setting are strengths of our study. In addition, participating centers have a long-standing expertise for CeAD, clinically and scientifically, which may have contributed to the high rate of complete datasets.^11,12,28^

However, the following important limitations should be considered. Referral bias is likely because data were derived from hospital-based cohorts at specialized centers, potentially leading to the inclusion of more women with high-risk features for outcome events. Furthermore, we did not investigate whether women were advised against pregnancy. Women at higher presumed recurrence risk may indeed have been discouraged from becoming pregnant and could therefore be underrepresented in the pregnancy group. These factors may limit the generalizability of our findings. Furthermore, due to the explorative nature of the study, we did not have central data monitoring or central adjudication of CeAD diagnoses, exposure variables, and outcome events, which limits data validity. Additionally, the small number of events leads to statistical imprecision, as demonstrated by the wide CIs, especially for individual outcome components, underscoring the need for cautious interpretation of our findings.

## Conclusions

In this cohort study of women who had had a prior CeAD, becoming pregnant was not associated with increased risks of recurrent CeAD, stroke, or death. These findings may be helpful for individual counseling and family planning for women with prior CeAD.

## Notes

Supplement 1. **eFigure 1.** Study flow chart **eFigure 2.** Sensitivity analysis 1 - Forest plot for main and secondary outcomes in women ≤ 42 years at timepoint of initial CeAD **eFigure 3.** Sensitivity analysis 2 - Forest plot for main and secondary outcomes in women ≤ 45 years at timepoint of initial CeAD **eFigure 4.** Sensitivity analysis 3 - Forest plot for main and secondary outcomes in women ≤ 49 years at timepoint of initial CeAD **eTable 1.** List of contributing centers **eTable 2.** Individual patient characteristics of women with recurrent CeAD associated with pregnancy **eTable 3.** Sensitivity analysis 1 - Baseline characteristics of women ≤ 42 years at timepoint of initial CeAD **eTable 4.** Sensitivity analysis 1 - Main and secondary outcomes of women ≤ 42 years at timepoint of initial CeAD **eTable 5.** Sensitivity analysis 2 - Baseline characteristics of women ≤ 45 years at timepoint of initial CeAD **eTable 6.** Sensitivity analysis 2 - Main and secondary outcomes of women ≤ 45 years at timepoint of initial CeAD **eTable 7.** Sensitivity analysis 3 - Baseline characteristics of women ≤ 49 years at timepoint of initial CeAD **eTable 8.** Sensitivity analysis 3 - Main and secondary outcomes of women ≤ 49 years at timepoint of initial CeAD **eTable 9.** Event rate during and outside pregnancy and postpartum of women with pregnancy during follow-up

Supplement 2.
Data Sharing Statement
