# Errors in Affiliations

**DOI:** 10.1001/jamanetworkopen.2025.32386

**Published:** 2025-08-20

In the Original Investigation titled “Long-Term Risk of Recurrent Cervical Artery Dissection and Stroke After Pregnancy,”^1^ published July 17, 2025, there were errors in 2 author affiliations. The affiliation for Alessandro Pezzini, MD, included “Stroke Unit Sanatorio Allende Cordoba, Argentina” but should have omitted that listing and been given as “Programma Stroke Care, Dipartimento di Emergenza-Urgenza, Azienda Ospedaliera Universitaria, Parma, Italia.” The affiliation for Rita Bella, MD, was “Dipartimento Di Scienze Mediche e Chirurgiche e Tecnologie Avanzate, Sezione di Neuroscienze, Università di Catania, Catania, Italy” but should have been “Dipartimento di Scienze Mediche, Chirurgiche e Tecnologie Avanzate, Università di Catania, Catania, Italy.” This article has been corrected.^1^

## References

[1] Fischer SK, Kaufmann JE, Metso TM, . Long-term risk of recurrent cervical artery dissection and stroke after pregnancy. JAMA Netw Open. 2025;8(7):e2521539. doi:10.1001/jamanetworkopen.2025.21539 40674050 PMC12272292

