# Supplementary material for: Long-Term Risk of Recurrent Cervical Artery Dissection and Stroke After Pregnancy
Source: JAMA Netw Open. 2025 Jul 17;8(7):e2521539. doi: 10.1001/jamanetworkopen.2025.21539 (PMC12272292; doi:10.1001/jamanetworkopen.2025.21539)
Supplement: Supplement 1. — eFigure 1. Study flow chart eFigure 2. Sensitivity analysis 1 - Forest plot for main and secondary outcomes in women ≤ 42 years at timepoint of initial CeAD eFigure 3. Sensitivity analysis 2 - Forest plot for main and secondary outcomes in women ≤ 45 years at timepoint of initial CeAD eFigure 4. Sensitivity analysis 3 - Forest plot for main and secondary outcomes in women ≤ 49 years at timepoint of initial CeAD eTable 1. List of contributing centers eTable 2. Individual patient characteristics of women with recurrent CeAD associated with pregnancy eTable 3. Sensitivity analysis 1 - Baseline characteristics of women ≤ 42 years at timepoint of initial CeAD eTable 4. Sensitivity analysis 1 - Main and secondary outcomes of women ≤ 42 years at timepoint of initial CeAD eTable 5. Sensitivity analysis 2 - Baseline characteristics of women ≤ 45 years at timepoint of initial CeAD eTable 6. Sensitivity analysis 2 - Main and secondary outcomes of women ≤ 45 years at timepoint of initial CeAD eTable 7. Sensitivity analysis 3 - Baseline characteristics of women ≤ 49 years at timepoint of initial CeAD eTable 8. Sensitivity analysis 3 - Main and secondary outcomes of women ≤ 49 years at timepoint of initial CeAD eTable 9. Event rate during and outside pregnancy and postpartum of women with pregnancy during follow-up [file jamanetwopen-e2521539-s001.pdf]

Supplemental Online Content

Fischer SK, Kaufmann JE, Metso TM, et al. Long-term risk of recurrent cervical artery dissection and stroke after pregnancy. *JAMA Netw Open*. 2025;8(7):e2521539. doi:10.1001/jamanetworkopen.2025.21539

eFigure 1. Study flow chart

eFigure 2. Sensitivity analysis 1 - Forest plot for main and secondary outcomes in women ≤ 42 years at timepoint of initial CeAD

eFigure 3. Sensitivity analysis 2 - Forest plot for main and secondary outcomes in women ≤ 45 years at timepoint of initial CeAD

eFigure 4. Sensitivity analysis 3 - Forest plot for main and secondary outcomes in women ≤ 49 years at timepoint of initial CeAD

eTable 1. List of contributing centers

eTable 2. Individual patient characteristics of women with recurrent CeAD associated with pregnancy

eTable 3. Sensitivity analysis 1 - Baseline characteristics of women ≤ 42 years at timepoint of initial CeAD

eTable 4. Sensitivity analysis 1 - Main and secondary outcomes of women ≤ 42 years at timepoint of initial CeAD

eTable 5. Sensitivity analysis 2 - Baseline characteristics of women ≤ 45 years at timepoint of initial CeAD

eTable 6. Sensitivity analysis 2 - Main and secondary outcomes of women ≤ 45 years at timepoint of initial CeAD

eTable 7. Sensitivity analysis 3 - Baseline characteristics of women ≤ 49 years at timepoint of initial CeAD

eTable 8. Sensitivity analysis 3 - Main and secondary outcomes of women ≤ 49 years at timepoint of initial CeAD

eTable 9. Event rate during and outside pregnancy and postpartum of women with pregnancy during follow-up

This supplemental material has been provided by the authors to give readers additional information about their work.

© 2025 Fischer SK et al. *JAMA Network Open.*

eFigure 1: Study flow chart

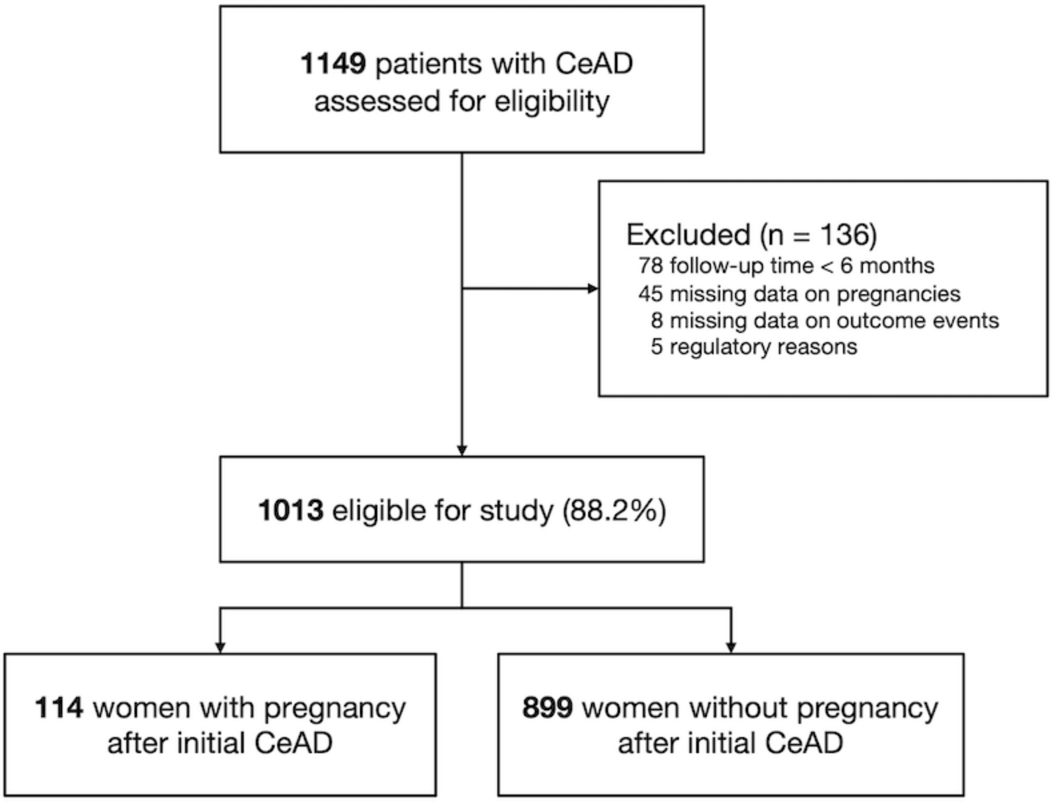

© 2025 Fischer SK et al. *JAMA Network Open.*

eFigure 2: Forest plot for main and secondary outcomes in women ≤ 42 years at timepoint of initial CeAD

Outcome event	With pregnancy, n (%)	No pregnancy, n (%)	Hazard Ratio (95% CI)
Composite outcome	9 (8.7)	43 (10.5)	0.82 (0.40-1.67)
adjusted for age			0.77 (0.36-1.64)
Recurrent dissection	6 (5.8)	23 (5.6)	1.03 (0.43-2.50)
adjusted for age			1.02 (0.39-2.69)
Ischemic stroke	2 (1.9)	15 (3.7)	0.61 (0.15-2.42)
adjusted for age			0.66 (0.15-2.87)
Intracerebral hemorrhage	1 (1.0)	3 (0.7)	1.65 (0.19-14.25)
adjusted for age			0.51 (0.05-4.76)
Death	0 (0.0)	3 (0.7)	0.65 (0.02-20.35)
adjusted for age			1.02 (0.03-36.49)

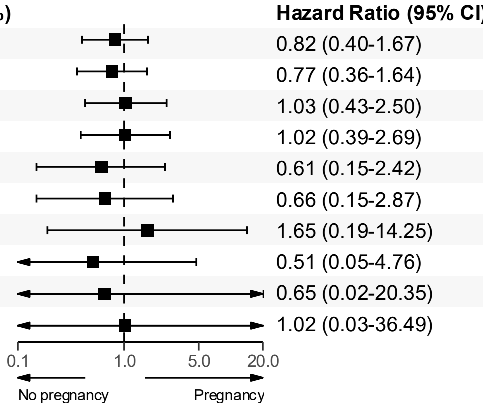

© 2025 Fischer SK et al. *JAMA Network Open.*

eFigure 3: Forest plot for main and secondary outcomes in women ≤ 45 years at timepoint of initial CeAD

Outcome event	With pregnancy, n (%)	No pregnancy, n (%)	Hazard Ratio (95% CI)
Composite outcome	9 (8.7)	49 (8.9)	0.93 (0.46-1.87)
adjusted for age			0.72 (0.34-1.54)
Recurrent dissection	6 (5.8)	27 (4.9)	1.14 (0.48-2.72)
adjusted for age			0.96 (0.37-2.50)
Ischemic stroke	2 (1.9)	17 (3.1)	0.69 (0.18-2.69)
adjusted for age			0.61 (0.14-2.62)
Intracerebral hemorrhage	1 (1.0)	3 (0.5)	2.10 (0.25-18.03)
adjusted for age			0.51 (0.05-4.79)
Death	0 (0.0)	3 (0.5)	0.78 (0.03-24.21)
adjusted for age			0.81 (0.02-30.43)

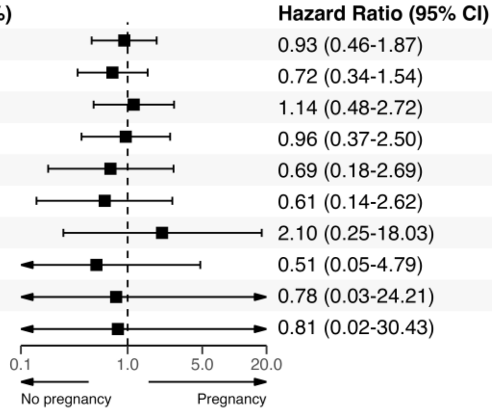

© 2025 Fischer SK et al. *JAMA Network Open.*

eFigure 4: Forest plot for main and secondary outcomes in women ≤ 49 years at timepoint of initial CeAD

Outcome event	With pregnancy, n (%)	No pregnancy, n (%)	Hazard Ratio (95% CI)
Composite outcome	10 (8.8)	55 (8.1)	1.00 (0.52-1.95)
adjusted for age			0.77 (0.38-1.57)
Recurrent dissection	7 (6.2)	29 (4.3)	1.37 (0.61-3.08)
adjusted for age			1.08 (0.45-2.63)
Ischemic stroke	2 (1.8)	20 (3.0)	0.63 (0.17-2.43)
adjusted for age			0.55 (0.13-2.25)
Intracerebral hemorrhage	1 (0.9)	3 (0.4)	2.26 (0.26-19.28)
adjusted for age			0.51 (0.05-4.81)
Death	0 (0.0)	4 (0.6)	0.62 (0.02-16.40)
adjusted for age			0.79 (0.03-19.71)

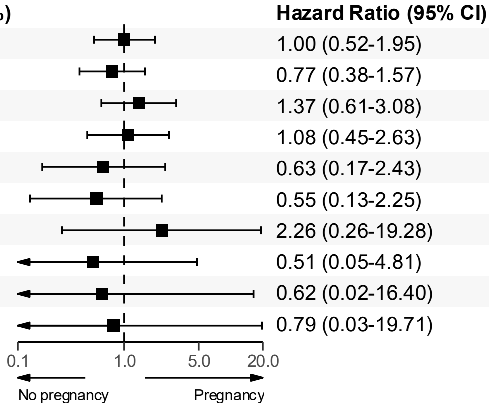

© 2025 Fischer SK et al. *JAMA Network Open.*

eTable 1: List of contributing centers

Country	Center	City	Time period of initial CeAD	Number of recruited patients
Switzerland	Department of Neurology and Stroke Center, Department of Clinical Research, University Hospital Basel and University of Basel	Basel	January 1997 – March 2023	130
	Department of Neurology, University Hospital Zurich and University of Zurich	Zurich	December 2014 – March 2022	32
Germany	Department for Neurology and experimental Neurology and Center for Stroke Research Berlin (CSB), Charité Universitätsmedizin, Berlin	Berlin	September 2013 – September 2021	18
	Department of Neurology, LMU University Hospital, LMU Munich	Munich	February 2014 – August 2021	10
Austria	Department of Neurology, Medical University of Innsbruck, Innsbruck	Innsbruck	March 1996 – February 2017	113
Argentina	Stroke Unit Sanatorio Allende, Cordoba	Córdoba	June 2005 – March 2022	31
Finland	Department of Neurology, Helsinki University Central Hospital, Helsinki	Helsinki	June 1994 – November 2008	63
Italy	IPSYS CEAD research collaboration	Brescia	January 2000 – June 2019	225
		Cagliari		8
		Catania		4
		Cremona		9
		Ferrara		9
		Genova		11
		La Spezia		4
		Mantova		19
		Messina		13
		Milano		25
		Padova		16
		Palermo		9
		Perugia		16
		Reggio Emilia		64
		Roma		22
		Rovigo		8
		San Benedetto		8
		Siena		6
		Torino		2
		Trento-Rovereto		5
		Verona		12
		Negrar		3
Israel	Hadassah-Hebrew University Medical Center, Jerusalem, Israel	Jerusalem	July 2010 – June 2022	39
Mexico	Stroke Clinic, Instituto Nacional de Neurología y Neurocirugía Manuel Velasco Suárez, Mexico City	Mexico-City	April 2000 – June 2021	63
USA	Department of Neurology, University of Utah, Salt Lake City	Salt Lake City	October 2010 – February 2023	22
	Department of Neurology, University of Virginia, Charlottesville	Charlottesville	May 1990 – April 2023	130

eTable 2: Individual patient characteristics of women with recurrent CeAD associated with pregnancy

Patient	Timepoint of recurrent CeAD	Clinical presentation of recurrent CeAD	Antithrombotic treatment at timepoint of recurrent CeAD	Mode of delivery before recurrent CeAD	Known vascular risk factors [a]	Mechanical trigger event prior to initial CeAD	Mechanical trigger event prior to recurrent CeAD
1	Postpartum	TIA	None	Vaginal	Hypercholesterolemia	No	Yes
2	Postpartum	Pure local symptoms	Clopidogrel	Cesarean	Hypercholesterolemia	No	No
3	Postpartum	Ischemic stroke	Aspirin	Vaginal	Active Smoking	No	Yes
4	Postpartum	Pure local symptoms	None	Vaginal	Former Smoking, Hypertension	No	Yes

Abbreviations: CeAD, cervical artery dissection; TIA, transient ischemic attack.
[a] Vascular risk factors obtained for the study were hypertension, hypercholesterolemia, smoking and diabetes. These factors were assessed at the time of the latest follow-up and not at the timepoint of recurrent CeAD.

© 2025 Fischer SK et al. *JAMA Network Open.*

eTable 3: Sensitivity analysis 1 - Baseline characteristics of women ≤ 42 years at timepoint of initial CeAD

	**All women ≤ 42 years** (n = 513)	**Women with pregnancy after initial CeAD** (n = 103)	**Women without pregnancy after initial CeAD** (n = 410)	**p-value unadjusted**	**OR unadjusted** (95% CI)
Age at initial CeAD (median [IQR])	35.00 [30.69, 39.00]	31.00 [27.00, 34.01]	36.00 [32.00, 39.00]	<0.001	NA
**Site of CeAD**					
Internal Carotid, n (%)	296 (57.8)	60 (58.3)	236 (57.7)	1.00	1.02 (0.66-1.59)
Vertebral, n (%)	249 (48.6)	50 (48.5)	199 (48.7)	1.00	1.00 (0.65-1.53)
Multivessel CeAD , n (%)	93 (18.1)	18 (17.5)	75 (18.3)	0.96	0.95 (0.52-1.64)
Occlusion , n (%)	151 (29.6)	31 (30.4)	120 (29.4)	0.94	1.05 (0.65-1.67)
**Clinical presentation at baseline**					
Pure local symptoms, n (%)	122 (23.8)	27 (26.2)	95 (23.2)	0.60	1.178 (0.71-1.92)
Ischemic stroke, n (%)	339 (66.1)	64 (62.1)	275 (67.1)	0.41	0.81 (0.52-1.27)
TIA, n (%)	71 (13.8)	13 (12.6)	58 (14.1)	0.81	0.88 (0.44-1.62)
**Predisposing factors**					
Mechanical trigger event[a], n (%)	110 (21.6)	31 (30.4)	79 (19.4)	0.02	1.81 (1.10-2.94)
Recent infection[a], n (%)	64 (13.0)	15 (15.3)	49 (12.4)	0.54	1.28 (0.66-2.34)
Migraine, n (%)	179 (35.0)	39 (38.2)	140 (34.2)	0.52	1.19 (0.76-1.86)
Connective tissue disease, n (%)	21 (4.3)	4 (4.3)	17 (4.4)	1.00	0.97 (0.27-2.70)
Associated to pregnancy, n (%)	23 (5.0)	5 (5.5)	18 (4.9)	1.00	1.13 (0.37-2.92)
**Vascular risk factors**					
Hypertension, n (%)	86 (16.8)	12 (11.7)	74 (18.1)	0.16	0.60 (0.30-1.11)
Diabetes, n (%)	11 (2.1)	1 (1.0)	10 (2.4)	0.59	0.39 (0.02-2.08)
Hypercholesterolemia, n (%)	48 (9.4)	7 (6.8)	41 (10.0)	0.41	0.65 (0.26-1.41)
Active smoker, n (%)	111 (21.7)	22 (21.4)	89 (21.8)	1.00	0.98 (0.57-1.63)

Abbreviations: CeAD, cervical artery dissection; NA, not applicable; OR, odds ratio; TIA, transient ischemic attack.
[a] in the month preceding the onset of initial CeAD.

© 2025 Fischer SK et al. *JAMA Network Open.*

eTable 4: Sensitivity analysis 1 - Main and secondary outcomes of women ≤ 42 years at timepoint of initial CeAD

	**All women ≤ 42 years** (n = 513)	**Women with pregnancy after initial CeAD** (n = 103)	**Women without pregnancy after initial CeAD** (n = 410)	**p-value unadjusted**	**HR unadjusted** (95% CI)	**HR adjusted for age**[a] (95% CI)
**Composite outcome**, n (%)	52 (10.1)	9 (8.7)	43 (10.5) [b]	0.73	0.82 (0.40-1.67)	0.77 (0.36-1.64)
**Recurrent dissection**, n (%)	29 (5.7)	6 (5.8)	23 (5.6) [b]	1.00	1.03 (0.43-2.50)	1.02 (0.39-2.69)
Asymptomatic, n (%)	4 (13.8)	0 (0.0)	4 (17.4)	0.55	NA	NA
Pure local symptoms, n (%)	15 (53.6)	3 (50.0)	12 (54.5)	1.00	NA	NA
Ischemic stroke, n (%)	6 (20.7)	1 (16.7)	5 (21.7)	1.00	NA	NA
TIA, n (%)	4 (13.8)	2 (33.3)	2 (8.7)	0.18	NA	NA
**Ischemic stroke**, n (%) (independent of recurrent CeAD)	17 (3.3)	2 (1.9)	15 (3.7)	0.54	0.61 (0.15-2.42)	0.66 (0.15-2.87)
**Intracerebral hemorrhage**, n (%)	4 (0.8)	1 (1.0)	3 (0.7) [b]	1.00	1.65 (0.19-14.25)	0.51 (0.05-4.76)
**Death**, n (%)	3 (0.6)	0 (0.0)	3 (0.7)	1.00	0.65 (0.02-20.35)	1.02 (0.03-36.49)
**Follow-up time in years**, median [IQR]	5.67 [2.25, 12.00]	7.00 [3.96, 11.04]	5.25 [2.00, 12.25]	0.09	NA	NA

Abbreviations: CeAD, cervical artery dissection; HR, hazard ratio; NA, not applicable; TIA, transient ischemic attack.
[a] Hazard ratios were adjusted for age at initial CeAD. [b] 1 patient in the non-pregnancy-group experienced two outcome events each (recurrent dissection plus intracerebral hemorrhage).

© 2025 Fischer SK et al. *JAMA Network Open.*

eTable 5: Sensitivity analysis 2 - Baseline characteristics of women ≤ 45 years at timepoint of initial CeAD

	**All women ≤ 45 years** (n = 655)	**Women with pregnancy after initial CeAD** (n = 104)	**Women without pregnancy after initial CeAD** (n = 551)	**p-value unadjusted**	**OR unadjusted** (95% CI)
Age at initial CeAD (median [IQR])	37.00 [32.00, 42.00]	31.00 [27.00, 34.27]	38.00 [34.00, 42.31]	<0.001	NA
**Site of CeAD**					
Internal Carotid, n (%)	390 (59.6)	61 (58.7)	329 (59.8)	0.91	0.95 (0.62-1.47)
Vertebral, n (%)	299 (45.7)	50 (48.1)	249 (45.3)	0.68	1.12 (0.73-1.70)
Multivessel CeAD , n (%)	114 (17.4)	18 (17.3)	96 (17.4)	1.00	0.99 (0.56-1.69)
Occlusion , n (%)	206 (31.6)	31 (30.1)	175 (31.9)	0.80	0.92 (0.57-1.44)
**Clinical presentation at baseline**					
Pure local symptoms, n (%)	150 (22.9)	27 (26.0)	123 (22.3)	0.49	1.22 (0.74-1.96)
Ischemic stroke, n (%)	439 (67.2)	65 (62.5)	374 (68.1)	0.31	0.78 (0.51-1.21)
TIA, n (%)	84 (12.8)	13 (12.5)	71 (12.9)	1.00	0.96 (0.49-1.76)
**Predisposing factors**					
Mechanical trigger event[a], n (%)	127 (19.6)	32 (31.1)	95 (17.4)	0.002	2.13 (1.32-3.40)
Recent infection[a], n (%)	77 (12.2)	15 (15.2)	62 (11.6)	0.41	1.36 (0.72-2.44)
Migraine, n (%)	238 (36.6)	39 (37.9)	199 (36.3)	0.85	1.07 (0.69-1.64)
Connective tissue disease, n (%)	25 (4.1)	4 (4.2)	21 (4.0)	1.00	1.04 (0.30-2.82)
Associated to pregnancy, n (%)	25 (4.2)	5 (5.4)	20 (4.0)	0.73	1.38 (0.45-3.52)
**Vascular risk factors**					
Hypertension, n (%)	115 (17.6)	12 (11.5)	103 (18.8)	0.10	0.56 (0.28-1.03)
Diabetes, n (%)	15 (2.3)	1 (1.0)	14 (2.6)	0.52	0.37 (0.02-1.87)
Hypercholesterolemia, n (%)	68 (10.4)	7 (6.7)	61 (11.2)	0.24	0.57 (0.23-1.21)
Active smoker, n (%)	147 (22.7)	22 (21.2)	125 (22.9)	0.79	0.90 (0.53-1.48)

Abbreviations: CeAD, cervical artery dissection; NA, not applicable; OR, odds ratio; TIA, transient ischemic attack.
[a] in the month preceding the onset of initial CeAD.

© 2025 Fischer SK et al. *JAMA Network Open.*

eTable 6: Sensitivity analysis 2 - Main and secondary outcomes of women ≤ 45 years at timepoint of initial CeAD

	**All women ≤ 45 years** (n = 655)	**Women with pregnancy after initial CeAD** (n = 104)	**Women without pregnancy after initial CeAD** (n = 551)	**p-value unadjusted**	**HR unadjusted** (95% CI)	**HR adjusted for age**[a] (95% CI)
**Composite outcome**, n (%)	58 (8.9) ‡	9 (8.7)	49 (8.9) [b]	1.00	0.93 (0.46-1.87)	0.72 (0.34-1.54)
**Recurrent dissection**, n (%)	33 (5.0)	6 (5.8)	27 (4.9) [b]	0.63	1.14 (0.48-2.72)	0.96 (0.37-2.50)
Asymptomatic, n (%)	5 (12.8)	0	5 (18.5)	0.56	NA	NA
Pure local symptoms, n (%)	19 (48.7)	3 (50.0)	13 (50.0)	1.00	NA	NA
Ischemic stroke, n (%)	10 (25.6)	1 (16.7)	7 (25.9)	1.00	NA	NA
TIA, n (%)	6 (15.4)	2 (33.3)	2 (7.4)	0.14	NA	NA
**Ischemic stroke**, n (%) (independent of recurrent CeAD)	19 (2.9)	2 (1.9)	17 (3.1)	0.75	0.69 (0.18-2.69)	0.61 (0.14-2.62)
**Intracerebral hemorrhage**, n (%)	4 (0.6)	1 (1.0)	3 (0.5) [b]	0.50	2.10 (0.25-18.03)	0.51 (0.05-4.79)
**Death**, n (%)	3 (0.5)	0	3 (0.5)	1.00	0.78 (0.03-24.21)	0.81 (0.02-30.43)
**Follow-up time in years**, median [IQR]	5.58 [2.17, 11.88]	7.04 [3.98, 11.33]	5.25 [2.00, 11.92]	0.04	NA	NA

Abbreviations: CeAD, cervical artery dissection; HR, hazard ratio; NA, not applicable; TIA, transient ischemic attack.
[a] Hazard ratios were adjusted for age at initial CeAD. [b] 1 patient in the non-pregnancy-group experienced two outcome events each (recurrent dissection plus intracerebral hemorrhage).

© 2025 Fischer SK et al. *JAMA Network Open.*

eTable 7: Sensitivity analysis 3 - Baseline characteristics of women ≤ 49 years at timepoint of initial CeAD

	**All women ≤ 45 years** (n = 790)	**Women with pregnancy after initial CeAD** (n = 113)	**Women without pregnancy after initial CeAD** (n = 677)	**p-value unadjusted**	**OR unadjusted** (95% CI)
Age at initial CeAD (median [IQR])	39.00 [33.00, 44.00]	31.00 [27.00, 36.00]	40.00 [35.00, 44.00]	<0.001	NA
**Site of CeAD**					
Internal Carotid, n (%)	487 (61.8)	67 (59.3)	420 (62.2)	0.63	0.88 (0.59-1.33)
Vertebral, n (%)	345 (43.8)	56 (49.6)	289 (42.8)	0.22	1.31 (0.88-1.96)
Multivessel CeAD , n (%)	136 (17.2)	21 (18.6)	115 (17.0)	0.78	1.12 (0.65-1.83)
Occlusion , n (%)	252 (32.1)	33 (29.5)	219 (32.5)	0.60	0.87 (0.55-1.33)
**Clinical presentation at baseline**					
Pure local symptoms, n (%)*	182 (23.0)	28 (24.8)	154 (22.7)	0.72	1.12 (0.69-1.76)
Ischemic stroke, n (%)*	521 (66.1)	71 (62.8)	450 (66.7)	0.49	0.85 (0.56-1.29)
TIA, n (%)*	110 (13.9)	18 (15.9)	92 (13.6)	0.61	1.20 (0.68-2.04)
**Predisposing factors**					
Mechanical trigger event [a], n (%)	145 (18.5)	34 (30.4)	111 (16.6)	<0.001	2.20 (1.39-3.43)
Recent infection [a], n (%)	93 (12.1)	15 (13.9)	78 (11.8)	0.65	1.20 (0.64-2.13)
Migraine, n (%)	297 (37.8)	42 (37.5)	255 (37.8)	1.00	0.99 (0.65-1.48)
Connective tissue disease, n (%)	33 (4.4)	5 (4.8)	28 (4.3)	1.00	1.11 (0.37-2.72)
Associated to pregnancy, n (%)	26 (3.6)	6 (5.9)	20 (3.2)	0.28	1.90 (0.68-4.60)
**Vascular risk factors**					
Hypertension, n (%)	142 (18.0)	14 (12.4)	128 (19.0)	0.12	0.60 (0.32-1.06)
Diabetes, n (%)	19 (2.4)	2 (1.8)	17 (2.5)	0.88	0.70 (0.11-2.47)
Hypercholesterolemia, n (%)	91 (11.6)	9 (8.0)	82 (12.2)	0.26	0.62 (0.28-1.22)
Active smoker, n (%)	172 (21.9)	25 (22.1)	147 (21.9)	1.00	1.01 (0.62-1.62)

Abbreviations: CeAD, cervical artery dissection; NA, not applicable; OR, odds ratio; TIA, transient ischemic attack.
[a] in the month preceding the onset of initial CeAD.

© 2025 Fischer SK et al. *JAMA Network Open.*

eTable 8: Sensitivity analysis 3 - Main and secondary outcomes of women ≤ 49 years at timepoint of initial CeAD

	**All women ≤ 45 years** (n = 790)	**Women with pregnancy after initial CeAD** (n = 113)	**Women without pregnancy after initial CeAD** (n = 677)	**p-value unadjusted**	**HR unadjusted** (95% CI)	**HR adjusted for age**[a] (95% CI)
**Composite outcome**, n (%)	65 (8.2)	10 (8.8)	55 (8.1) [b]	0.940	1.00 (0.52-1.95)	0.77 (0.38-1.57)
**Recurrent dissection**, n (%)	36 (4.6)	7 (6.2)	29 (4.3) [b]	0.335	1.37 (0.61-3.08)	1.08 (0.45-2.63)
Asymptomatic, n (%)	5 (13.9)	0 (0.0)	5 (17.2)	0.559	NA	NA
Pure local symptoms, n (%)	18 (51.4)	3 (42.9)	15 (53.6)	0.691	NA	NA
Ischemic stroke, n (%)	9 (25.0)	2 (28.6)	7 (24.1)	1.000	NA	NA
TIA, n (%)	5 (13.9)	3 (42.9)	2 (6.9)	0.040	NA	NA
**Ischemic stroke**, n (%) (independent of recurrent CeAD)	22 (2.8)	2 (1.8)	20 (3.0)	0.757	0.63 (0.17-2.43)	0.55 (0.13-2.25)
**Intracerebral hemorrhage**, n (%)	4 (0.5)	1 (0.9)	3 (0.4) [b]	0.461	2.26 (0.26-19.28)	0.51 (0.05-4.81)
**Death**, n (%)	4 (0.5)	0 (0.0)	4 (0.6)	1.000	0.62 (0.02-16.4)	0.79 (0.03-19.71)
**Follow-up time in years**, median [IQR]	5.42 [2.10, 11.71]	7.17 [3.92, 12.00]	5.17 [2.00, 11.50]	0.013	NA	NA

Abbreviations: CeAD, cervical artery dissection; HR, hazard ratio; NA, not applicable; TIA, transient ischemic attack.
[a] Hazard ratios were adjusted for age at initial CeAD. [b] 1 patient in the non-pregnancy group experienced two outcome events each (recurrent dissection plus intracerebral hemorrhage).

© 2025 Fischer SK et al. *JAMA Network Open.*

eTable 9: Event rate during and outside pregnancy and postpartum of women with pregnancy during follow-up

	During pregnancy and postpartum period	Outside pregnancy and postpartum period
Events	4 recurrent CeADs (postpartum)<br>1 ischemic stroke (postpartum)	3 recurrent CeADs<br>1 ischemic stroke<br>1 hemorrhagic stroke
Follow-up time[a]	157.57 patient-years	793.10 patient-years
Event rate	3.17% per patient-year	0.63% per patient-year

[a] Individual data on pregnancy duration are unavailable. We assumed a standard pregnancy duration of 40 weeks, plus an additional 12 weeks for the postpartum period to calculate the follow-up time for the pregnancy and postpartum period.

© 2025 Fischer SK et al. *JAMA Network Open.*
