# Supplementary material for: Long-Term Risk of Recurrent Cervical Artery Dissection and Stroke After Pregnancy
Source: JAMA Netw Open. 2025 Jul 17;8(7):e2521539. doi: 10.1001/jamanetworkopen.2025.21539 (PMC12272292; doi:10.1001/jamanetworkopen.2025.21539)
Supplement: Supplement 2. — Data Sharing Statement [file jamanetwopen-e2521539-s002.pdf]

Data Sharing Statement

Fischer. Long-Term Risk of Recurrent Cervical Artery Dissection and Stroke After Pregnancy. *JAMA Netw Open*. Published July 17, 2025. doi:10.1001/jamanetworkopen.2025.21539

Data
Data available: No

Additional Information
Explanation for why data not available: Study data can be made available on reasonable request to the corresponding author. Such requests must be accompanied by detailed study proposals, a description of study objectives, and a statistical analysis plan. Each request will be checked for compatibility with regulatory (ethics committee) requirements as well as compatibility with patient informed consent.
